# Supplementary material for: Healthcare seeking behavior and antibiotic use for diarrhea among children in rural Bangladesh before seeking care at a healthcare facility
Source: Sci Rep. 2025 Jul 20;15:26359. doi: 10.1038/s41598-025-09479-w (PMC12277439; doi:10.1038/s41598-025-09479-w)
Supplement: Supplementary file 1 — Supplementary Information. [file 41598_2025_9479_MOESM1_ESM.docx]

**Supplementary Information**


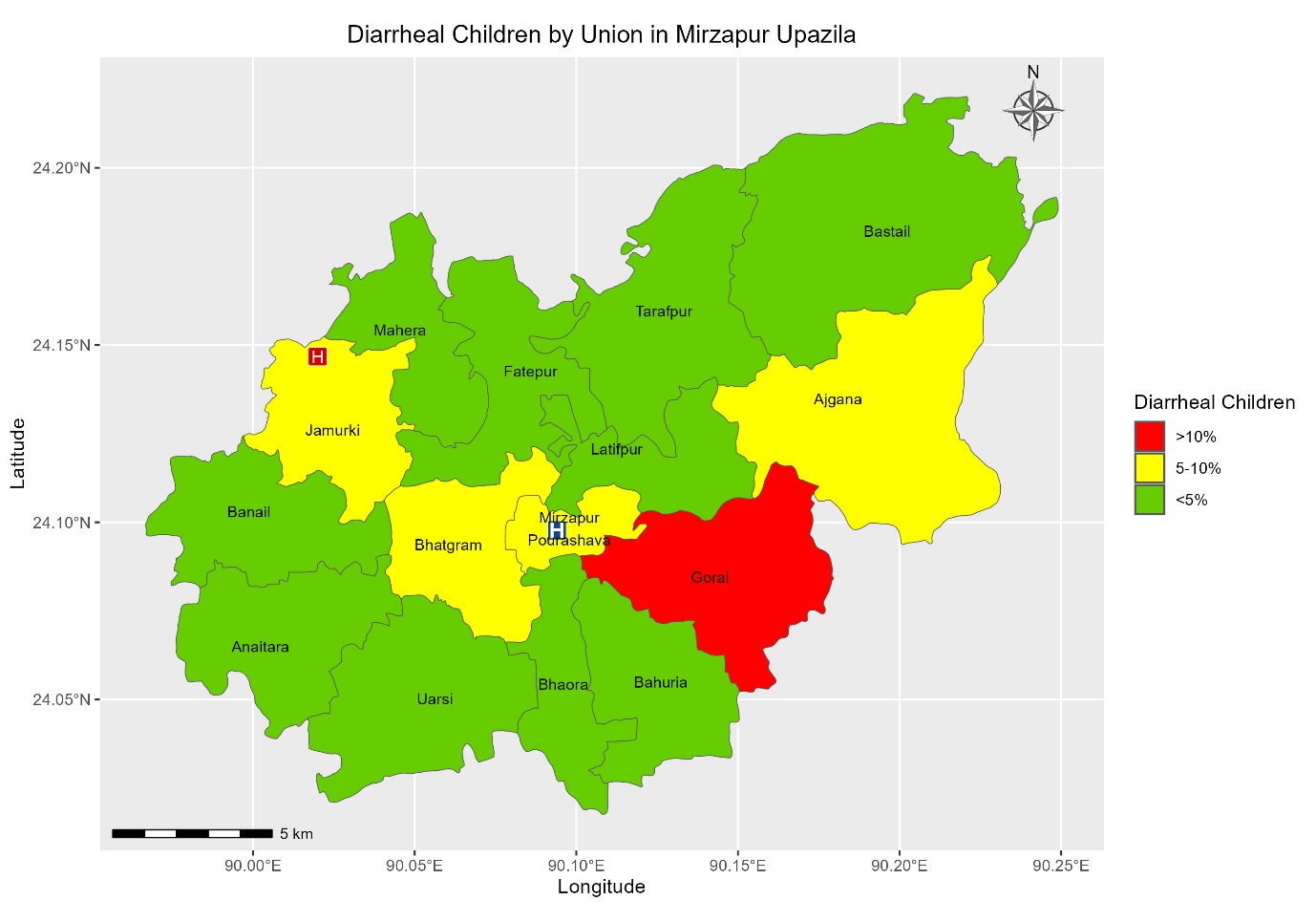


Figure S1a. Union-wise distribution of the under-5 diarrheal children who visited Kumudini Hospital. The map is generated using [R version 4.4.1](https://cran.r-project.org/bin/windows/base/old/4.4.1/), along with the “ggplot2” and “sf” packages.

The proportion of children with diarrhea per union was calculated as children with diarrhea from a union divided by the total children with diarrhea.


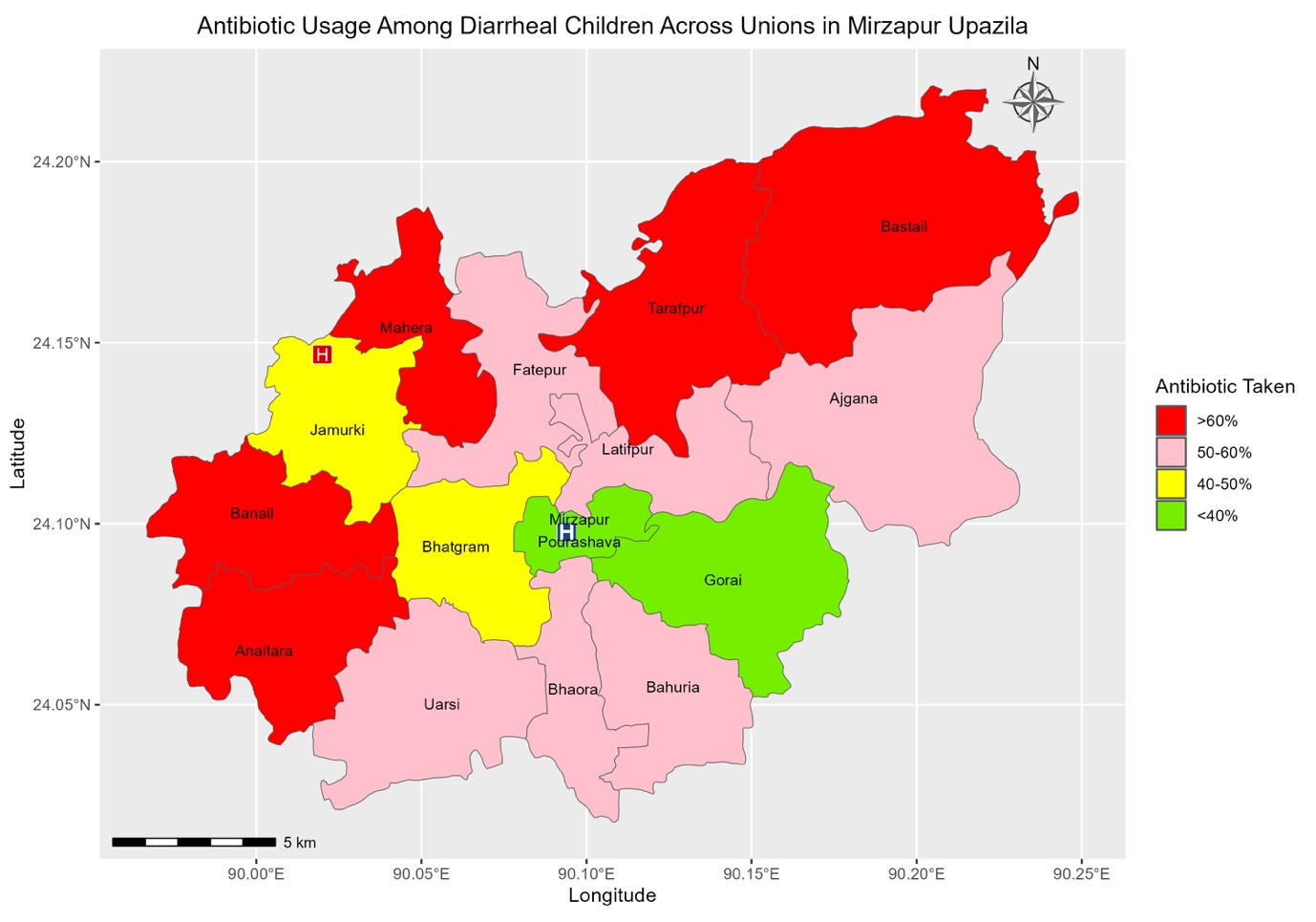


Figure S 1b. Union-wise distribution of the proportion of children who received antibiotics before visiting Kumudini Hospital. The map is generated using [R version 4.4.1](https://cran.r-project.org/bin/windows/base/old/4.4.1/), along with the “ggplot2” and “sf” packages.

The proportion of children who received antibiotics from each union was calculated by the number of diarrheal children who received antibiotics from that union, divided by the total number of diarrheal children who visited Kumudini Hospital from the same union.
